# Vitamin D Deficiency and Obsessive–Compulsive Disorder Severity: A Cross-Sectional Study

**DOI:** 10.3390/life16061002

**Published:** 2026-06-14

**Authors:** Donatella Marazziti, Federico Mucci, Matteo Gambini, Enrico Fazio, Leonardo Cazzato, Manuel Glauco Carbone, Riccardo Gurrieri

**Affiliations:** 1 Department of Clinical and Experimental Medicine, Section of Psychiatry, University of Pisa, 57, Via Roma, 56100 Pisa, Italy; gambinimatteo1996@gmail.com (M.G.); e.fazio2@gmail.com (E.F.); leocazzato@gmail.com (L.C.); 2 Department of Mental Health and Addiction, Azienda Unità Sanitaria Locale (USL) Toscana Nord Ovest, 42, Via Paolini, 55100 Lucca, Italy; 3 Department of Medicine and Surgery, Division of Psychiatry, University of Insubria, 9, Via Guicciardini, 21100 Varese, Italy; manuelglaucocarbone@gmail.com (M.G.C.); riccardo.gurrieri@santannapisa.it (R.G.); 4 Health Science Interdisciplinary Center, Sant’Anna School of Advanced Studies, 33, Piazza Martiri della Libertà, 56127 Pisa, Italy

**Keywords:** obsessive–compulsive disorder, vitamin D, 25-hydroxyvitamin D, Yale–Brown obsessive–compulsive scale, symptom severity, age at onset, cross-sectional study

## Abstract

Obsessive–compulsive disorder (OCD) is a chronic and disabling psychiatric condition whose neurobiological underpinnings remain incompletely characterized. A growing body of evidence suggests that vitamin D, through its modulatory actions on neuroinflammation, serotonin synthesis, and cortico-striato-thalamo-cortical circuitry, may be implicated in its clinical expression. The present cross-sectional study examined the association between serum 25-hydroxyvitamin D levels and OCD severity in 306 adult outpatients with a diagnosis of OCD, of whom 173 had vitamin D measurements available. Symptom severity was assessed through the Yale–Brown Obsessive–Compulsive Scale (Y-BOCS), and associations were examined using non-parametric tests, partial correlations and multivariable linear regression adjusted for age, gender, age at onset, and bipolar comorbidity. Mean vitamin D was 20.0 ± 13.1 ng/mL, with 60.1% of patients meeting criteria for deficiency. Lower vitamin D levels correlated inversely with Y-BOCS total score (ρ = −0.26, *p* = 0.001) and with both subscales, and deficient patients showed a mean Y-BOCS total approximately 5.5 points higher than non-deficient ones. In multivariable models, lower vitamin D (β = −0.253, *p* = 0.001) and earlier age at onset (β = −0.278, *p* = 0.001) independently predicted greater severity (R^2^ = 0.133), while a history of suicide attempts neither predicted severity nor moderated the vitamin D association. These findings support vitamin D status as a biological correlate of OCD severity and warrant longitudinal and interventional investigation.

## 1. Introduction

Obsessive–compulsive disorder (OCD) is a chronic and disabling psychiatric condition characterized by the recurrence of intrusive and unwanted thoughts, images or impulses (obsessions), which generate marked anxiety and distress, and by repetitive behaviours or mental acts (compulsions) that the patient feels driven to perform in order to neutralize the obsessive content or to prevent dreaded outcomes [1,2]. Although insight into the irrational nature of these phenomena is generally preserved, the voluntary control that patients exert over them is poor, with the consequence that the disorder typically interferes with social, occupational and family functioning to a degree that has been compared to that of other severe psychiatric conditions, including schizophrenia and bipolar disorder [1]. Population-based epidemiological studies have estimated the lifetime prevalence of OCD at approximately 2–3% and the twelve-month prevalence at approximately 1.1% in the general adult population [3,4], with figures that have been substantively confirmed by the World Mental Health Surveys conducted in over thirty countries [5], and a moderate but consistent female preponderance has been documented in adulthood [6]. The disorder is currently classified among obsessive–compulsive and related disorders in the Diagnostic and Statistical Manual of Mental Disorders, Fifth Edition, Text Revision (DSM-5-TR), and in the International Classification of Diseases, Eleventh Revision (ICD-11), reflecting its distinctive phenomenological and neurobiological profile [7,8].

The phenomenology of OCD is notoriously heterogeneous, since the contents of obsessions and the topographies of compulsions may vary considerably across patients. A long tradition of factor-analytic and meta-analytic work has progressively converged on the identification of a small number of stable symptom dimensions, which include contamination/cleaning, checking, symmetry/ordering, hoarding and the so-called taboo cluster of aggressive, sexual and religious obsessions [9,10,11]. A second source of clinical heterogeneity in OCD is represented by the age at onset of the disorder, which has long been recognized as a key prognostic variable and has motivated the proposal of two clinically distinct subtypes, one characterized by an early onset (typically before adulthood) and the other by a later onset [12]. Patients with early-onset OCD tend to display a greater familial loading, a more severe symptomatic profile, a higher comorbidity with tic disorders and a less favourable response to first-line treatments [13,14], while those with later onset more frequently present with affective comorbidity and with a relatively less severe symptom burden [15]. The neurobiological underpinnings of OCD have been progressively clarified by the convergence of genetic, neurochemical and neuroimaging evidence, all of which point to a complex disturbance of cortico-striato-thalamo-cortical circuitry [16]. Twin and family studies have estimated the heritability of OCD at approximately 40–50%, with higher figures for early-onset cases, and recent large-scale genome-wide association analyses have shown that the genetic architecture of the disorder is polygenic, with multiple common and rare variants of small effect contributing to overall risk [17,18]. At the neurochemical level, although the long-standing serotonergic hypothesis remains supported by the clinical efficacy of selective serotonin reuptake inhibitors as first-line pharmacotherapy, an increasing body of work has implicated glutamatergic dysregulation in the pathophysiology of the disorder, with alterations in glutamate concentrations having been documented in the orbitofrontal cortex and the basal ganglia by means of magnetic resonance spectroscopy [19,20]. These observations have motivated the exploration of glutamate-modulating agents as potential augmentation strategies in treatment-resistant patients [21]. Structural neuroimaging meta-analyses have consistently reported volumetric reductions in the orbitofrontal cortex and in the anterior cingulate cortex, alongside volumetric increases in the caudate nuclei [22]. Again, functional studies have shown hyperactivation of the anterior cingulate cortex during error-processing tasks [23], aberrant resting-state connectivity between fronto-parietal and fronto-striatal networks [24], and altered limbic activation during emotional tasks [25]. Taken together, these observations support a model in which OCD is conceptualized as a disorder of the balance between goal-directed and habitual control of behaviour, with compulsivity emerging as a transdiagnostic dimension that the disorder shares with other neuropsychiatric conditions such as substance use disorders and behavioural addictions [26].

Within this multifactorial framework, the relationship between OCD and suicidal behaviour has attracted growing scientific attention over the past two decades, since the conventional view of OCD as a low-suicide-risk anxiety disorder has been progressively challenged by an accumulating body of clinical and epidemiological evidence. A meta-analysis based on more than fifty studies has estimated that approximately 27% of patients with OCD experience lifetime suicidal ideation and that approximately 13% have engaged in at least one suicide attempt during the course of their illness [27], and earlier work had likewise reported lifetime ideation rates of around 47% and lifetime attempt rates of around 11% [28], with figures that consistently exceed those observed in the general population. The reasons for this risk are likely multifactorial, since they involve a combination of dimensional, comorbidity-related and trait-level vulnerabilities, and have prompted calls for the systematic integration of suicide risk assessment into the routine clinical management of patients with OCD [29,30].

In parallel with these clinical developments, the role of vitamin D as a potential biomarker and modulator of psychiatric disease has emerged as a topic of considerable interest. Indeed, vitamin D, beyond its classical role in calcium homeostasis and bone metabolism, exerts a wide range of neurobiological actions, which include the modulation of neuroinflammatory processes, the regulation of neurotransmitter synthesis, and a contribution to neurodevelopmental and neuroprotective mechanisms [31]. At the molecular level, the existence of vitamin D response elements within the promoter region of the TPH2 gene, which encodes the brain-specific isoform of tryptophan hydroxylase, provides a plausible mechanism by which adequate vitamin D status may regulate the central synthesis of serotonin, while a parallel repressive action on the TPH1 gene located outside the blood–brain barrier may exert opposite effects on peripheral serotonin synthesis [32]. The relevance of this mechanism has been extended to a broader range of neuropsychiatric conditions, including attention-deficit/hyperactivity disorder, autism spectrum disorders, bipolar disorder, schizophrenia, and impulsive behaviour [33], and is consistent with the widespread distribution of vitamin D receptors throughout brain regions involved in emotional regulation, cognitive control and behavioural inhibition.

Despite the plausibility of these mechanistic links and the potential clinical relevance of vitamin D status as a modifiable biological variable, the empirical literature specifically examining the relationship between vitamin D and obsessive–compulsive disorder remains relatively limited, and is largely confined to a small number of clinical studies conducted on heterogeneous samples. A pilot investigation by our group on a sample of fifty adult outpatients with OCD reported that virtually all participants displayed vitamin D concentrations below the conventional sufficiency threshold of 30 ng/mL and that vitamin D values were inversely correlated with Y-BOCS total scores and with the compulsion subscale, with some sex-related peculiarities [34]. Pediatric studies have yielded broadly convergent, albeit not unanimous, findings, since some documented significantly lower vitamin D levels in children and adolescents with OCD compared with healthy controls together with a negative correlation between vitamin D and symptom severity [35], while others failed to find a statistically significant difference between affected and unaffected children [36]. The available evidence, therefore, suggests that vitamin D may be implicated in the clinical expression of obsessive–compulsive symptomatology, but the comparatively small sample sizes of previous studies, the heterogeneity of their methodological approaches, and the relative scarcity of investigations carried out on adult clinical populations leave several aspects of the relationship between vitamin D and OCD severity insufficiently characterized, particularly in real-world clinical settings in which patients with substantial psychiatric comorbidity and complex pharmacological regimens are typically encountered.

The present study was designed to examine, in a large clinical sample of adult outpatients with a primary DSM-5-TR diagnosis of obsessive–compulsive disorder, the cross-sectional association between serum vitamin D levels and obsessive–compulsive symptom severity, as measured by the Yale–Brown Obsessive–Compulsive Scale [37], with the further aim of exploring whether age at onset of the disorder represents an independent correlate of severity over and above demographic and clinical confounders. Given the well-documented relevance of suicidal behaviour as a clinical dimension of OCD, the study additionally evaluated, on an exploratory basis, whether the relationship between vitamin D and obsessive–compulsive severity might differ between patients with and without a lifetime history of suicide attempts, in order to assess whether vitamin D status interacts with suicidal vulnerability in shaping the clinical expression of the disorder.

## 2. Materials and Methods

### 2.1. Study Design and Participants

The present investigation was conducted as a cross-sectional, observational study at an outpatient clinic specializing in obsessive–compulsive and related disorders, that is the Section of Psychiatry of the Department of Clinical and Experimental Medicine at the University of Pisa (Italy), where consecutive adult outpatients of both sexes between 18 and 60 years old were screened for eligibility. Patients were considered eligible when they met DSM-5-TR criteria for a primary diagnosis of obsessive–compulsive disorder (OCD), with the diagnosis established by senior psychiatrists with extensive experience in obsessive–compulsive spectrum conditions and confirmed through the structured assessment further described. Comorbid psychiatric conditions were permitted, provided that OCD remained the principal reason for clinical referral, since the aim of the study was to characterize the clinical and biological correlates of OCD severity within a real-world clinical population rather than within an artificially purified diagnostic group.

Exclusion criteria were: presence of major medical diseases that could either interfere with psychiatric assessment or potentially affect vitamin D metabolism (chronic kidney or liver disease, malabsorption syndromes, disorders of calcium–parathyroid metabolism, active malignancy, severe autoimmune or inflammatory diseases, and severe neurological disorders), substance abuse, intake of vitamin D and pregnancy in women.

All participants received a detailed verbal and written explanation of the aims and procedures of the study before being asked to sign informed consent, and the study, approved by the Ethics Committee at Pisa university (Protocol #.295/36), was conducted in accordance with the principles of the Declaration of Helsinki and its subsequent amendments.

The final analytic sample comprised 306 patients with a primary DSM-5-TR diagnosis of OCD, recruited in the period November 2024–October 2025, of whom 173 had serum vitamin D measurements available; the discrepancy between the total sample and the vitamin D subsample reflects the retrospective availability of laboratory data within the clinical records and is addressed in the Limitations Section.

The design and reporting of the present investigation conform to the Strengthening the Reporting of Observational Studies in Epidemiology (STROBE) recommendations for cross-sectional studies.

### 2.2. Diagnostic and Clinical Assessment

The psychiatric diagnosis of each participant was established through a clinical interview conducted by senior psychiatrists and structured according to the Mini-International Neuropsychiatric Interview (M.I.N.I.) [38], an instrument that, by virtue of its standardized format and its alignment with DSM and ICD criteria, has long been used in both clinical and research settings to ensure diagnostic reliability across raters. Beyond the principal diagnosis of OCD, the M.I.N.I. was systematically administered to detect comorbid psychiatric conditions, with particular attention paid to mood disorders—and especially to bipolar disorder, given its known clinical relevance in obsessive–compulsive populations and its inclusion as a covariate in subsequent multivariable models.

Socio-demographic and clinical information was collected through a structured anamnestic interview conducted at the time of enrolment, and included age, sex, marital status, educational attainment, occupational status, age at onset of obsessive–compulsive symptoms, mode of onset (acute versus progressive), illness course (chronic versus episodic), history of perinatal trauma, presence of obsessive–compulsive personality traits, lifetime history of suicide attempts, current and previous psychopharmacological treatments, and previous exposure to cognitive behavioural therapy. When available, body mass index (BMI), date of blood sampling, and information on current vitamin D supplementation were also extracted from clinical records. The season of blood sampling was derived from the date of serum 25-hydroxyvitamin D measurement and categorized according to standard calendar seasons.

The severity of obsessive–compulsive symptomatology was quantified by means of the Yale–Brown Obsessive–Compulsive Scale (Y-BOCS) [37], which is widely regarded as the reference instrument for the assessment of OCD severity and which yields, through its ten clinician-rated items scored from 0 (no symptoms) to 4 (severe symptoms), a total score ranging from 0 to 40, together with two subscale scores of equal range that capture, respectively, the obsessive and the compulsive components of the disorder. In the present study the Y-BOCS total score served as the principal measure of clinical severity, while the obsession and compulsion subscales were used as secondary outcomes in those analyses where the differential involvement of the two symptom dimensions was of specific interest.

### 2.3. Vitamin D Measurement

Serum 25-hydroxyvitamin D concentrations were determined as part of the routine biochemical workup performed at the recruiting centre. Ten mL of venous blood was drawn from all fasting and sitting subjects between 8 and 9 a.m. The blood was then transferred to plastic tubes (BD Vacutainer, Becton, Dickinson and Company, Franklin Lakes, NJ, USA) for vitamin D measurements by the common clinical-chemical method of competitive protein-binding (CPB) assay using the LIAISON 25 OH Vitamin D TOTAL Assay (DiaSorin, Saluggia, Italy).

Vitamin D values are expressed throughout the manuscript in nanograms per millilitre (ng/mL), and patients were classified, in accordance with the most widely adopted clinical thresholds, into three categories that correspond, respectively, to vitamin D deficiency (serum levels below 20 ng/mL), insufficiency (levels comprised between 20 and 29.9 ng/mL), and sufficiency (levels equal to or above 30 ng/mL).

### 2.4. Statistical Analysis

Continuous variables were summarized as mean and standard deviation, complemented by median and range whenever the underlying distribution departed substantially from normality, while categorical variables were described in terms of absolute frequencies and percentages; the assumption of normality was assessed by graphical inspection of the distributions in conjunction with the Kolmogorov–Smirnov and Shapiro–Wilk tests, and, given the predominantly non-Gaussian behaviour of the principal clinical and laboratory variables, non-parametric methods were preferentially adopted for bivariate analyses.

Bivariate associations between obsessive–compulsive severity, expressed as the Y-BOCS total score and its obsession and compulsion subscales, and continuous or ordinal predictors such as vitamin D levels and age at onset were examined by means of Spearman’s rank-order correlation coefficient (ρ); to assess the robustness of the principal associations and to control for potential demographic and clinical confounders, partial correlation analyses were subsequently performed first by adjusting for age and sex and then by additionally adjusting for the presence of comorbid bipolar disorder. Group comparisons between two independent categories were carried out by means of the Mann–Whitney U test for continuous outcomes and through the χ^2^ test, or Fisher’s exact test where the expected cell frequencies were small, for categorical outcomes; these comparisons were applied, among others, to the contrast between patients with and without a lifetime history of suicide attempts, between those with and without comorbid bipolar disorder, and between vitamin D categories defined according to clinical thresholds. When predictors were articulated into more than two ordered or unordered categories, group differences in Y-BOCS scores were tested through the Kruskal–Wallis test, with Bonferroni-adjusted pairwise post hoc comparisons performed in the event of a significant overall result; the presence of monotonic dose–response trends across ordered categories of vitamin D was further verified by means of the Jonckheere–Terpstra test, which is particularly suited to the detection of ordered alternatives in non-parametric settings.

To identify independent correlates of obsessive–compulsive severity, multivariable linear regression models were fitted with the Y-BOCS total score as the principal dependent variable and, in secondary analyses, with the obsession and compulsion subscale scores as additional outcomes; the core model included serum vitamin D levels, age at onset, age, sex, and bipolar comorbidity as predictors, and a hierarchical regression strategy was additionally used to assess the incremental contribution of vitamin D over and above demographic and clinical covariates. Regression results were reported in the form of unstandardized (B) and standardized (β) coefficients, together with their associated 95% confidence intervals and significance values, and the overall fit of each model was evaluated through the coefficient of determination (R^2^), the adjusted R^2^, and the omnibus F test, while multicollinearity was monitored through tolerance values and variance inflation factors.

In order to test whether the relationship between vitamin D and OCD severity differed according to a lifetime history of suicide attempts, a separate linear regression model was fitted in which a vitamin D × suicide attempt interaction term was included alongside the principal effects of vitamin D, age at onset, suicide attempt history, age, sex, and bipolar comorbidity. With a view to limiting the influence of extreme laboratory values on the regression estimates, sensitivity analyses were carried out using a trimmed vitamin D variable that excluded values above 80 ng/mL, and the possibility of a non-linear relationship between vitamin D and obsessive–compulsive severity was further explored by introducing a quadratic vitamin D term into the regression models.

Missing data were handled in a manner appropriate to each analytical context, with available-case analysis adopted for descriptive statistics and non-parametric comparisons, pairwise deletion adopted for Spearman correlations, and listwise deletion adopted for the regression and partial correlation models; the effective analytic sample size therefore varied across analyses, particularly for those involving laboratory measurements, and is reported alongside the corresponding results. All statistical tests were two-tailed, the threshold of significance was set at *p* < 0.05, and all analyses were performed using IBM SPSS Statistics, version 25.0 (IBM Corp., Armonk, NY, USA).

## 3. Results

### 3.1. Sample Characteristics

The final sample comprised 306 adult outpatients with a primary DSM-5-TR diagnosis of obsessive–compulsive disorder, whose sociodemographic and core clinical profile is summarized in Table 1. The cohort consisted predominantly of young adults, with a mean age of 33.4 ± 11.6 years and a modest male preponderance (58.8%), and displayed a relatively early mean age at onset of 18.7 ± 6.9 years. The course of illness was mainly chronic rather than episodic, the mode of onset was more frequently progressive than acute, and a substantial proportion of participants presented obsessive–compulsive personality traits. A lifetime history of suicide attempts was present in 16 patients (5.2%). Psychiatric comorbidity was the rule rather than the exception, since nearly nine out of ten participants met criteria for at least one additional psychiatric diagnosis, and bipolar disorder was by far the most frequent comorbid condition, affecting 29.7% of the cohort, with other affective and anxiety disorders represented at markedly lower rates; medical comorbidities were comparatively infrequent, with thyroid disorders being the most common (Table 2). To evaluate the representativeness of the vitamin D subsample, patients with available serum 25-hydroxyvitamin D measurements (*n* = 173) were compared with those without available vitamin D data (*n* = 133) on key demographic and clinical variables. No significant differences emerged between the two groups in age, sex distribution, Y-BOCS total score or prevalence of bipolar comorbidity (all *p* > 0.05), suggesting that the vitamin D subsample was broadly comparable to the remaining cohort with respect to the main variables relevant to the present analyses.

### 3.2. Treatment Profile

Pharmacological treatment patterns reflected the clinical complexity of the cohort (Table 3). Approximately half of the patients were receiving polypharmacy, defined as the concurrent prescription of two or more psychotropic agents, and pharmacological augmentation strategies, operationalized as the addition of an antipsychotic or a mood stabilizer to an antidepressant backbone, were employed in more than a third of the sample. Selective serotonin reuptake inhibitors represented the most frequently prescribed class, in keeping with their status as first-line pharmacotherapy for the disorder, while second-generation antipsychotics, valproate and lithium constituted the most common augmentation agents, and cognitive behavioural therapy had been offered to approximately one fifth of participants.

### 3.3. OCD Severity and Symptom Profile

The cohort exhibited a moderate-to-severe level of obsessive–compulsive symptomatology, with a mean Y-BOCS total score of 25.7 ± 8.1 and a broadly balanced contribution from the obsession and compulsion subscales (Table 4). The phenomenological profile was dominated by time consumption, distress, and impaired control over symptoms, rather than by loss of insight, and the dimensional structure was markedly multifaceted, since 82.3% of participants presented three or more concurrent symptom dimensions, with checking, miscellaneous intrusive phenomena and aggressive obsessions emerging as the most prevalent domains. Greater dimensional burden showed a modest positive association with symptom severity at the bivariate level, but the number of current symptom dimensions did not remain an independent predictor of Y-BOCS total score once demographic and clinical covariates were taken into account, indicating that dimensional complexity in this cohort functions as a marker of broader clinical heterogeneity rather than as an autonomous determinant of symptom severity.

### 3.4. Vitamin D Levels and Their Association with OCD Severity

Serum 25-hydroxyvitamin D concentrations were available for 173 of the 306 participants, and the distribution of values pointed to a pronounced tendency toward hypovitaminosis D within the cohort, with a mean of 20.0 ± 13.1 ng/mL and with 60.1% of the subsample meeting the conventional threshold for vitamin D deficiency (<20 ng/mL), 24.9% falling within the insufficiency range (20–29.9 ng/mL) and only 15.0% attaining sufficient levels (≥30 ng/mL). Spearman correlation analyses disclosed statistically significant inverse associations between serum vitamin D and obsessive–compulsive severity across all three Y-BOCS indices, namely the total score (ρ = −0.26, *p* = 0.001), the obsession subscale (ρ = −0.28, *p* < 0.001) and the compulsion subscale (ρ = −0.24, *p* = 0.001), and these associations persisted with virtually unchanged magnitude after partialling out age and gender and after additionally controlling for bipolar comorbidity (Table 5). The pattern was confirmed when vitamin D was analyzed as a categorical variable, since patients in the deficient range displayed a mean Y-BOCS total score of 29.2 ± 7.7 (*n* = 104), compared with 22.3 ± 7.3 among those with insufficient levels (*n* = 43) and 25.9 ± 9.5 among those with sufficient levels (*n* = 26), with the Kruskal–Wallis test yielding a highly significant overall difference (H = 21.71, *p* < 0.001); pairwise post hoc comparisons indicated that the principal contrast lay between the deficient and the insufficient strata, whereas differences between the insufficient and the sufficient strata, and between the deficient and the sufficient strata, did not reach statistical significance. The numerically intermediate mean observed in the sufficient stratum, which is non-monotonic relative to the adjacent insufficient category, likely reflects the limited size and the comparatively broad dispersion of the sufficient subgroup (SD = 9.5 on only 26 cases), and is consistent with the Jonckheere–Terpstra test for an ordered alternative, which rests on rank-based pairwise comparisons across classes and which, in the present analysis, clearly supported a monotonic trend of decreasing symptom severity with increasing vitamin D class (standardized J-T = −3.84, *p* < 0.001). When deficient and non-deficient patients were compared directly through an independent-samples test, the former exhibited a mean Y-BOCS total score approximately 5.5 points higher than the latter (29.2 ± 7.7 versus 23.7 ± 8.3; t = −4.47, *p* < 0.001), indicating that the clinically meaningful threshold within this cohort is situated at the boundary between deficiency and non-deficiency rather than within the non-deficient range itself. Sensitivity analyses in which vitamin D values exceeding 80 ng/mL were trimmed yielded virtually identical correlation coefficients, and the introduction of a quadratic vitamin D term into the regression models did not reveal any evidence of a non-linear relationship (*p* = 0.711), further supporting the interpretation of the association as predominantly linear, or alternatively threshold-like, rather than curvilinear. Bivariate comparisons between patients with and without comorbid bipolar disorder did not disclose significant differences in Y-BOCS total, obsession or compulsion scores (all *p* > 0.83), a result that, while not in itself modifying the choice of bipolar comorbidity as a covariate in the subsequent multivariable models, provides a post hoc reassurance that the association between vitamin D and obsessive–compulsive severity is unlikely to be confounded by affective comorbidity.

### 3.5. Multivariable Analyses and the Vitamin D × Suicide Attempt Interaction

The core multivariable linear regression model, which included trimmed vitamin D levels, age at onset, age, gender and bipolar comorbidity as predictors of the Y-BOCS total score, identified lower vitamin D levels (β = −0.253, *p* = 0.001) and earlier age at onset (β = −0.278, *p* = 0.001) as the only two statistically significant independent correlates of greater obsessive–compulsive severity, while neither age nor gender nor bipolar comorbidity emerged as a significant predictor; the overall model was significant (F = 6.24, *p* < 0.001) and accounted for 13.3% of the variance in the Y-BOCS total score A separate linear regression model was then fitted in order to evaluate whether the relationship between vitamin D and symptom severity differed according to a lifetime history of suicide attempts, and in this model vitamin D (β = −0.217, *p* = 0.004) and age at onset (β = −0.280, *p* = 0.001) retained their independent associations with severity, whereas suicide attempt status itself (*p* = 0.475) and the vitamin D × suicide attempt interaction term (*p* = 0.670) were both clearly non-significant (overall model F(7,161) = 3.26, *p* = 0.003, R^2^ = 0.124), indicating that the association between vitamin D and obsessive–compulsive severity did not differ between patients with and without a history of suicide attempts. Consistent with this negative interaction, direct bivariate comparisons between patients with and without suicide attempts did not reveal significant differences in Y-BOCS total (25.7 ± 8.2 versus 25.4 ± 6.2; *p* = 0.975), obsession (*p* = 0.764) or compulsion (*p* = 0.870) scores, and only two nominal differences emerged at the symptom-dimension level, namely a lower prevalence of contamination obsessions (χ^2^ = 4.07, *p* = 0.044) and of the contamination–checking cluster (χ^2^ = 4.14, *p* = 0.042) among patients with a history of suicide attempts, neither of which would survive a Bonferroni correction for the fourteen dimensional comparisons performed (adjusted threshold *p* < 0.0036). Taken together, these analyses indicate that, within the limits of the small number of patients with a lifetime history of suicide attempts (*n* = 16), suicidal vulnerability did not emerge as a clinical dimension capable of modifying the relationship between vitamin D status and obsessive–compulsive severity, and that the association between lower vitamin D levels and greater symptom burden appears to operate independently of this particular risk marker.

## 4. Discussion

### 4.1. Principal Findings

The present cross-sectional investigation, conducted in a large naturalistic sample of 306 adult outpatients with OCD, disclosed three convergent observations that provide a coherent biological reading of clinical severity in this condition. First, serum 25-hydroxyvitamin D concentrations were inversely associated with Y-BOCS total and subscale scores across the entire range of analyses, from non-parametric bivariate correlations to partial correlations adjusted for demographic and clinical confounders and to multivariable regression models, with a standardized coefficient of approximately −0.25 that remained stable across statistical approaches. Second, patients meeting the conventional threshold for vitamin D deficiency (<20 ng/mL) exhibited a mean Y-BOCS total score approximately 5.5 points higher than their non-deficient counterparts, a difference that, in the context of the widely adopted definition of a clinically significant treatment response as a 25–35% reduction in Y-BOCS from baseline [39], appears clinically meaningful. Third, earlier age at onset of the disorder emerged as an independent correlate of greater obsessive–compulsive severity, with an effect size comparable to that of vitamin D, supporting the view that developmental and biological determinants may converge in shaping the clinical expression of OCD. The overall model accounted for approximately 13% of the variance in Y-BOCS total score, a modest figure, consistent with the multifactorial architecture of a disorder in which genetic, neurodevelopmental, neurochemical and environmental factors all play partially overlapping roles [1,16,21], and to which the two correlates identified here are likely to contribute only a fraction of the full pathophysiological picture.

### 4.2. Vitamin D and Obsessive–Compulsive Severity

The inverse relationship between serum vitamin D levels and Y-BOCS scores documented in the present cohort aligns, both qualitatively and in magnitude, with the clinical evidence that has examined vitamin D status in obsessive–compulsive populations. A previous pilot investigation from our group on fifty adult outpatients reported that virtually all participants displayed vitamin D values below the conventional sufficiency threshold and that these values were inversely correlated with Y-BOCS scores, with some sex-related peculiarities [34]. A more recent case–control study on 174 drug-naïve adult patients with OCD and 170 healthy controls reported significantly lower vitamin D concentrations in the clinical group, together with substantially stronger inverse correlations between vitamin D and Y-BOCS total, obsession and compulsion scores [40]. Complementary evidence from a comprehensive diagnostic screening protocol applied to an inpatient OCD population documented suboptimal vitamin D levels in approximately three quarters of the patients assessed [41], whereas the paediatric literature has yielded partially convergent but not unanimous findings [35,36,42]. In the present sample the pattern observed at the categorical level was non-monotonic, since the sufficient stratum (*n* = 26) displayed a numerically higher mean Y-BOCS total score than the insufficient stratum. This observation likely reflects the limited size and the broad dispersion of the sufficient subgroup (SD = 9.5) rather than a genuine reversal of the dose–response relationship, and the 5.5-point difference between deficient and non-deficient patients, together with the significance of the Jonckheere–Terpstra trend test, suggests that the association is better characterized as a threshold-like effect clustered around the 20 ng/mL cut-off than as a strictly linear dose–response gradient across the full range of serum concentrations. The high overall prevalence of hypovitaminosis D observed in our cohort (60.1% below 20 ng/mL) is, moreover, compatible with the figures reported in epidemiological surveys of the Italian general adult population, in which vitamin D insufficiency has been described as endemic even in geographical areas with favourable solar irradiation [43,44]. However, the absence of an internal healthy control group precludes any direct comparison of absolute vitamin D values between OCD and non-OCD subjects.

### 4.3. Neurobiological Plausibility

The biological plausibility of an association between vitamin D status and OC severity rests on partially overlapping mechanistic considerations, each supported by independent experimental evidence. The inflammatory hypothesis of OCD has gained increasing support, with neuroimaging evidence documenting increased translocator protein distribution volumes in cortico-striato-thalamo-cortical circuits and an association between orbitofrontal inflammatory signal and Y-BOCS distress-related items [45]. Systematic reviews of peripheral cytokine alterations, monocyte activation and autoimmune comorbidities have added further, if heterogeneous, support [46,47,48,49]. Since vitamin D exerts well-characterized actions on both innate and adaptive immunity [31], deficiency may represent a plausible contributor to the immune-inflammatory burden associated with more severe obsessive–compulsive symptomatology. The role of vitamin D biology, however, is not confined to immune signalling. Functional vitamin D response elements identified within the promoter region of the brain-specific tryptophan hydroxylase 2 gene, together with a parallel repressive element in the peripheral TPH1 gene, have been shown to provide a molecular substrate through which adequate vitamin D status may be required for the normal synthesis of brain serotonin while simultaneously restraining peripheral serotonin production [32,33], and more recent cellular work has documented that 1,25-dihydroxyvitamin D also down-regulates the serotonin transporter and monoamine oxidase-A in serotonergic neuronal lines [50]. Given the centrality of the serotonergic system both to the pathophysiology of OCD and to the efficacy of the selective serotonin reuptake inhibitors as first-line pharmacotherapy, it appears reasonable, if far from proven, to hypothesize that inadequate vitamin D levels might contribute to a suboptimal tone of central serotonergic transmission and, indirectly, to clinical severity. Anatomically, vitamin D receptors and the activating enzyme CYP27B1 are directly expressed in brain regions developmentally and functionally implicated in OCD, with detailed immunohistochemical mapping having documented widespread VDR expression throughout the human brain and particularly high density in the substantia nigra, hippocampus and cortical areas involved in executive control [51,52,53]. This distribution substantially overlaps with the cortico-striato-thalamo-cortical network whose dysregulation has been repeatedly implicated in OCD [21,22,26]. A neurotrophic dimension is also plausible, as vitamin D has been shown to influence brain-derived neurotrophic factor expression [54], and adjunctive strategies such as vitamin D and N-acetylcysteine have been discussed as potential avenues in treatment-resistant OCD [55]. These converging lines of evidence render the possibility that the observed association between lower vitamin D and higher obsessive–compulsive severity represents a biologically coherent convergence plausible enough to warrant dedicated mechanistic and interventional research.

### 4.4. Age at Onset and the Developmental Interface

The independent predictive role of earlier age at onset for greater obsessive–compulsive severity is consistent with a long-standing literature that has repeatedly identified age at onset as one of the most consistent prognostic variables in OCD, with early-onset cases displaying a greater familial loading, a more severe and chronic symptomatic profile, a higher comorbidity with tic disorders and a less favourable response to first-line treatments [12,13,14,15,56]. Investigations in large international samples confirmed that early-onset subjects typically exhibit longer durations of untreated illness, poorer insight and more complex comorbidity profiles than their late-onset counterparts [57]. The coexistence, in the present sample, of earlier age at onset and lower vitamin D as independent correlates of symptom severity invites a developmental interpretation. If vitamin D plays, as the experimental literature increasingly suggests, a genuine role in the ontogeny of cortical and subcortical circuits relevant to emotional regulation and behavioural control, with both developmental and early-life vitamin D status having been associated with the later risk of several neuropsychiatric conditions [51,58], then one might wonder whether insufficient vitamin D status during the neurodevelopmental windows in which obsessive–compulsive symptoms typically first emerge could represent an additional, potentially modifiable contributor to the early establishment of the disorder, in interaction with the genetic and environmental vulnerabilities that have been more extensively characterized in the literature [16,18]. The concurrent emergence of earlier onset and lower vitamin D as independent correlates of severity may be compatible with a framework in which developmental and biological determinants interact rather than operate in parallel.

### 4.5. Suicide Attempts and Symptom Severity

Lifetime suicide attempt history did not predict obsessive–compulsive severity, nor did it moderate the vitamin D-severity association—a result to be read against the extensive literature that has documented elevated rates of suicidal ideation and behaviour in OCD and a substantial excess risk of death by suicide when compared with the general population [27,28,29,30,59,60,61,62,63]. The apparent discrepancy may be more a question of statistical power than of substantive contradiction, since only 16 patients in our cohort reported a lifetime history of suicide attempts, a number that constrains the ability of our analyses to detect the modest main effects on Y-BOCS scores and any moderation effect. The pattern observed seems, moreover, consistent with the converging evidence that suicidal behaviour in OCD depends more on comorbidity-related factors, trait-level impulsivity and dimensional features of the disorder, such as taboo and aggressive obsessions, than on raw symptom severity [64,65]. Furthermore, the observation that vitamin D status did not differentiate patients with and without a lifetime history of suicide attempts is suggestive that the association between vitamin D and OCD severity may operate independently of this particular vulnerability marker, a pattern that will need to be re-examined in larger samples.

### 4.6. Limitations

Several limitations should be considered when interpreting the findings of the present study. First, the cross-sectional design does not allow any causal inference to be drawn regarding the relationship between vitamin D status and obsessive–compulsive severity, and it remains entirely possible that low vitamin D represents a consequence rather than a cause of severe symptomatology, possibly mediated by reduced outdoor activity, impaired dietary habits or the indirect metabolic consequences of chronic stress; longitudinal observational studies and, ideally, randomized interventional trials will be required to disentangle the direction of effect and to ascertain whether correction of vitamin D deficiency might translate into clinically meaningful reductions in obsessive–compulsive symptom burden. Second, serum vitamin D measurements were available for 173 of the 306 participants, reflecting the retrospective availability of laboratory data within the clinical records rather than a prespecified biomarker protocol, so that a degree of selection bias cannot be formally excluded and the generalizability of the vitamin D findings to the broader OCD population should be considered with caution. Associations were confirmed after adjustment for BMI, season of sampling and supplementation. Third, although information on body mass index, date of blood sampling and current vitamin D supplementation was extracted from clinical records when available, these variables were not incorporated into the core multivariable models because of incomplete availability and because current vitamin D supplementation represented an exclusion criterion. Therefore, residual confounding by determinants of vitamin D status, including body mass index, season of blood sampling, sunlight exposure, dietary intake and physical activity, cannot be excluded. Fourth, serum 25-hydroxyvitamin D was measured using a competitive protein-binding assay, which is less precise than liquid chromatography–tandem mass spectrometry and some modern immunoassays. This methodological aspect may have affected absolute vitamin D estimates and, consequently, the classification of patients across deficiency, insufficiency and sufficiency categories. Fifth, the study did not include a healthy control group, which precludes any direct inferential claim that patients with OCD are more vitamin D-deficient than the background Italian population, in which hypovitaminosis D has been consistently reported at similarly high rates [43,44]. Also, the modest male predominance observed in the present sample also differs from the slight female preponderance reported in epidemiological studies of OCD. This discrepancy may reflect referral patterns and the characteristics of a tertiary outpatient clinic specializing in obsessive–compulsive spectrum conditions, and should therefore be considered when generalizing the findings to community-based OCD populations. Sixth, the prevalence of comorbid bipolar disorder in our cohort (29.7%) is substantially higher than the 6–10% typically reported in international primary-OCD samples [66,67,68], a feature that reflects the tertiary specialization of the recruiting centre in obsessive–compulsive spectrum conditions and that limits the generalizability of the present findings to less selected OCD populations. Finally, the multivariable model explained a modest proportion of the variance in Y-BOCS total score, consistent with the multidimensional determinants of OCD severity and with the realistic scope of single biological markers in clinical characterization.

## 5. Conclusions

In this cohort of adult outpatients with OCD, the present study documents an inverse association between serum 25-hydroxyvitamin D concentrations and obsessive–compulsive symptom severity, which remains stable across non-parametric, partial-correlation and multivariable-regression approaches and which is mirrored by a clinically meaningful difference in Y-BOCS total scores between patients with and without vitamin D deficiency. Earlier age at onset emerges, in parallel, as an independent correlate of greater severity, while a lifetime history of suicide attempts does not predict severity or moderate the vitamin D-severity association within the power constraints of the relevant sub-sample. These findings support serum vitamin D status as a biological correlate of OCD severity, although longitudinal and interventional studies are required to clarify directionality and clinical relevance.

## Figures and Tables

**Table 1:** Sociodemographic and clinical characteristics of the study sample (*N* = 306).

Variable	*N*	Mean ± SD or *n* (%)
Age, years	306	33.42 ± 11.63
Female sex	306	126 (41.2)
Married	273	68 (24.9)
Employed	270	164 (60.7)
Elementary education	270	1 (0.4)
Secondary education	270	20 (7.4)
High school	270	145 (53.7)
University	270	101 (37.4)
Postgraduate	270	3 (1.1)
Age at onset, years	264	18.66 ± 6.92
Chronic course	268	224 (83.6)
Acute onset	267	81 (30.3)
Perinatal trauma	267	78 (29.2)
OC personality traits	267	157 (58.8)
Lifetime suicide attempts	306	16 (5.2)

Values are mean ± SD for continuous variables and *n* (%) for categorical variables. *N* = participants with available data. OC, obsessive–compulsive; SD, standard deviation.

**Table 2:** Psychiatric and medical comorbidities (*N* = 306).

Variable	*n* (%)
No psychiatric comorbidity	34 (11.1)
One psychiatric comorbidity	176 (57.5)
Two psychiatric comorbidities	96 (31.4)
Bipolar disorder	91 (29.7)
Generalized anxiety disorder	26 (8.5)
Persistent depressive disorder	20 (6.5)
Social anxiety disorder	9 (2.9)
Psychotic spectrum disorder	4 (1.3)
Panic disorder	3 (1.0)
Borderline personality disorder	2 (0.7)
Other psychiatric disorders	213 (69.6)
No medical comorbidity	243 (87.4)
Thyroid disorders	13 (4.7)
Cardiovascular disorders	5 (1.8)
Female genital disorders	5 (1.8)
Gastrointestinal disorders	4 (1.4)
Neurological disorders	3 (1.1)

Values are *n* (%). Patients could present more than one comorbid condition.

**Table 3:** Current pharmacological treatments and therapeutic strategies (*N* = 306).

Variable	*n* (%)
No psychotropic medication	54 (17.6)
Monotherapy	102 (33.3)
Polypharmacy (≥2)	150 (49.0)
SSRIs	157 (51.3)
Clomipramine	34 (11.1)
SNRIs	29 (9.5)
Other antidepressants	13 (4.2)
Other tricyclics	8 (2.6)
Second-generation antipsychotics	35 (13.0)
First-generation antipsychotics	13 (4.8)
Valproate	47 (17.5)
Lithium	33 (12.3)
Other mood stabilizers	20 (7.4)
Gabapentinoids	37 (13.8)
Benzodiazepines	98 (32.0)
Pharmacological augmentation	110 (35.9)
Cognitive behavioural therapy	62 (20.3)
SSRI augmentation	60 (19.6)

Polypharmacy: ≥2 psychotropic medications. Augmentation: antipsychotic or mood stabilizer added to antidepressant backbone. SSRI, selective serotonin reuptake inhibitor; SSRIs, selective serotonin reuptake inhibitors; SNRIs, serotonin–noradrenaline reuptake inhibitors.

**Table 4:** Obsessive–compulsive symptom severity and dimensional characteristics.

Variable	*N*	Mean ± SD or %
Y-BOCS total	269	25.69 ± 8.13
Y-BOCS obsessions	270	13.06 ± 4.01
Y-BOCS compulsions	270	12.68 ± 4.45
Low dimensional burden (0–2)	—	17.6%
Moderate (3–5)	—	40.5%
High (≥6)	—	41.8%
Current OCD dimensions	—	5.67 ± 2.33

Y-BOCS total 0–40; subscales 0–20; higher = greater severity. OCD, obsessive–compulsive disorder; SD, standard deviation; Y-BOCS, Yale–Brown Obsessive–Compulsive Scale.

**Table 5:** (**a**) Spearman correlations between serum vitamin D and Y-BOCS scores (*N* = 171). (**b**) Y-BOCS total across vitamin D clinical categories.

**(a)**		
**Y-BOCS Index**	**ρ**	***p*** **-Value**
Total	−0.26	0.001
Obsessions	−0.28	<0.001
Compulsions	−0.24	0.001
**(b)**		
**Vitamin D Category**	***n***	**Y-BOCS Total (Mean ± SD)**
Deficiency (<20 ng/mL)	104	29.18 ± 7.66
Insufficiency (20–29.9)	43	22.29 ± 7.34
Sufficiency (≥30)	26	25.85 ± 9.47

ρ, Spearman rank-order correlation. Y-BOCS, Yale–Brown Obsessive–Compulsive Scale. Correlation analyses were based on 171 participants because two patients with available serum 25-hydroxyvitamin D measurements had missing Y-BOCS data. Kruskal–Wallis H = 21.71, *p* < 0.001. Jonckheere–Terpstra standardized J-T = −3.84, *p* < 0.001. Post hoc: principal contrast deficient vs. non-deficient. For the dichotomous comparison, pooled non-deficient patients (≥20 ng/mL) showed a mean Y-BOCS total of 23.65 ± 8.34 (t = −4.47, *p* < 0.001).

## Data Availability

The data supporting the findings of this study are not publicly available due to privacy, ethical and legal restrictions related to the use of sensitive clinical information. De-identified and aggregated data may be made available by the corresponding author upon reasonable request, subject to approval by the relevant Ethics Committee and in accordance with institutional policies and applicable data protection regulations.

## Abbreviations

The following abbreviations are used in this manuscript:

25(OH)D	25-hydroxyvitamin D
BMI	Body mass index
CBT	Cognitive behavioural therapy
CPB	Competitive protein-binding
CSTC	Cortico-striato-thalamo-cortical
DSM-5-TR	Diagnostic and Statistical Manual of Mental Disorders, Fifth Edition, Text Revision
ICD-11	International Classification of Diseases, Eleventh Revision
M.I.N.I.	Mini-International Neuropsychiatric Interview
OCD	Obsessive–compulsive disorder
OC	Obsessive–compulsive
SD	Standard deviation
SNRI	Serotonin–noradrenaline reuptake inhibitor
SSRI	Selective serotonin reuptake inhibitor
STROBE	Strengthening the Reporting of Observational Studies in Epidemiology
TPH1	Tryptophan hydroxylase 1
TPH2	Tryptophan hydroxylase 2
VDR	Vitamin D receptor
Y-BOCS	Yale–Brown Obsessive–Compulsive Scale
